# COVID-19 and Neurologic Manifestations: An Experience at Major New York City Hospitals

**DOI:** 10.7759/cureus.24049

**Published:** 2022-04-11

**Authors:** Subhadra Nori, Alberto Moran, Joseph Franolich, Jasal Patel, Michelle Stern

**Affiliations:** 1 Rehabilitation Medicine, Elmhurst Hospital Center, Queens, USA; 2 Rehabilitation Medicine, Queens Hospital Center, Queens, USA; 3 Quality, Queens Hospital Center, Queens, USA; 4 Rehabilitation Medicine, Montefiore Medical Center, Bronx, USA; 5 Rehabilitation Medicine, Jacobi Medical Center, Bronx, USA

**Keywords:** anosmia, cognitive dysfunction, guillian barre'syndrome, encephalopathy, seizures, motor weakness, ataxia, cerebro vascular disease, neurological manifestations, covid19

## Abstract

Introduction: The exact incidence of neurologic manifestations in coronavirus disease 2019 (COVID-19) patients is not clear. The New York City Hospital system has been severely affected by the COVID-19 pandemic between December 2019 and 202. A large number of patients were treated at these centers. This study aims to investigate the incidence of such neurologic manifestations. Secondly, we wanted to find out if there is a correlation between comorbidities and neurologic manifestations in patients with COVID-19.

Methodology: A retrospective analysis of 5,455 Electronic Medical Records of patients with a positive polymerase chain reaction (PCR) result admitted to Elmhurst Hospital, Queens Hospital Center, Jacobi Medical Center, and North Central Bronx Hospitals, four of the 11 teaching hospitals in the NYCHH (New York Health + Hospitals) between 3-1-2020 to 8-31-2020 was carried out. Comprehensive data were collected using medical documentation in five categories: demographic details, comorbidities, symptoms, laboratory findings, and radiologic examinations. All neurologic manifestation keywords were provided to the statisticians by two trained and board-certified physiatrists. Neurologic manifestations were categorized into central nervous system (CNS) manifestations and peripheral nervous system (PNS) manifestations.

Results; Out of the 5,455 patients, 285 patients (5.2%) had central nervous system manifestations, a prevalence in people older than 50, and had a high incidence of comorbidities. We found increased D-dimer and high C reactive protein levels. Our findings agree with two of the three authors with significant case volume.

## Introduction

The New York City Health and Hospitals (NYCH+H) consists of public hospitals and clinics. It is the most extensive municipal health system in the United States. It serves 1.4 million patients, of whom 475,000 are uninsured. More than 190 languages are spoken; This corporation operates 11 acute care hospitals, five nursing homes, six diagnostic and treatment centers, and more than 70 community-based primary care centers.

New York City (NYC) was an epicenter of the coronavirus disease 2019 (COVID-19) outbreak in the United States in spring 2020 [1]. In March-May 2020. Corinne N. Thompson et al. in their report stated that according to the CDC (Center for Disease Control ), approximately 203,000 laboratory-confirmed COVID-19 cases were reported to the NYC Department of Health and Mental Hygiene (DOHMH), during the first three months of the pandemic. The crude fatality rate among confirmed cases was 9.2% overall and 32.1% among hospitalized patients. Incidence, hospitalization rates, and mortality were highest among Black/African American and Hispanic/Latino persons, as well as those who were living in neighborhoods with high poverty, aged ≥75 years, and with underlying medical conditions. The highest rates of cases, hospitalizations, and deaths were concentrated in communities of color, high-poverty areas, and among persons aged ≥75 years or with underlying conditions. The crude fatality rate was 9.2% overall and 32.1% among hospitalized patients A majority of these cases were admitted to the city hospitals due to their socioeconomic status.

NYC had an average of 27,000 people per square mile, according to the 2010 census. That is more than double the density of Chicago and Philadelphia and more than three times the density of Los Angeles. Housing conditions in New York City have one of the nation's most complicated housing markets. These conditions are more likely to impact immigrants, making them renters and causing affordability problems. Multivariate analyses reveal that foreign-born renters are more likely to live in overcrowded housing. Coupled with the high number of immigrants in New York, these factors may be why NYC became the epicenter of COVID -19. These four teaching hospitals were chosen for this study because they served the highest concentration of immigrants and were most heavily affected during the pandemic. Two hospitals were in the borough of Bronx and two in Queens and made up the most diversified neighborhoods in the United States.

The COVID-19 outbreak quickly became a pandemic posing several epidemiological, social, and medical issues. Healthcare systems and governments worldwide were forced to take extreme measures to contain the infection and develop treatments. Du et al. reported 85 fatal cases in the city of Wuhan in Hubei province, China [2]. The median age of the patients was 59 years, with higher morbidity and mortality among the elderly and those with coexisting conditions (similar to the situation with influenza); 56% of the patients were male and 44% female. The first report on neurologic manifestations of COVID-19 was reported from Wuhan, China by Mao et al. [3 ].

Our retrospective study included 5,455 hospitalized patients with laboratory confirmation of SARS-CoV-2 analyzed patients diagnosed as having COVID-19; the aim was to analyze the incidence of neurological manifestations in patients affected by COVID-19 and to compare our findings with those already published. 

At the beginning of the COVID-19 pandemic, the overall situation was utterly unprecedented. We faced several challenges Many patients were rapidly deteriorating. To avoid cross-infection during the outbreak, we had to minimize visitations, thus, limiting our capacity to obtain history and background information from the family of those patients who were unable to provide such information. Examinations were difficult since there was minimal patient participation in many cases. Therefore, the diagnosis of nervous system manifestations mainly depended on patients' subjective symptoms. Specific diagnostic testing tools such as lumbar puncture, MRI, and electrodiagnosis had to be selective due to the fear of cross-contamination. Many patients developed altered mental status, seizures, and encephalopathy-like symptoms. Disseminated intravascular clotting (DIC) was a standard feature. Acute cerebrovascular disease includes ischemic Stroke, cerebral hemorrhage diagnosed by clinical symptoms, and head CT (computed tomography). Seizure diagnosis was based on clinical symptoms.

## Materials and methods

The study was performed according to the BRANY approved study design principles. Institutional Review Board (IRB) approvals were obtained from the local hospitals via the H+H board (BRANY IRB File # 21-12-028-373 {HHC}) BRANY (Biomedical Research Alliance of New York) is a national organization that supports sponsors and investigators involved in research in a wide variety of therapeutic areas, medical devices, biological and diagnostic trials.

A confirmed case of COVID-19 was defined as a positive result with a Real-time Reverse Transcriptase Polymerase-Chain-Reaction (RT-PCR) assay using a nasopharyngeal swab. Radiologic assessments included chest and head CT and all laboratory testing (a complete blood cell count, blood chemical analysis, coagulation testing, assessment of liver and renal function, C-reactive protein, creatine kinase, and lactate dehydrogenase) was performed according to the clinical care needs of the patient. 5,455 Electronic Medical Records were reviewed retrospectively. Comprehensive data collection was done using medical documentation in five categories: demographic details, comorbidities, symptoms, laboratory findings, and radiologic examinations. Demographics details included age, sex, ethnicity, and comorbidities including hypertension, diabetes, cardiac or cerebrovascular disease, malignancy, and chronic kidney disease, symptoms from the hospital admission fever, cough, anorexia, diarrhea, throat pain, abdominal pain, nervous system symptoms, laboratory findings, and CT scan (chest and head), MRI (when performed). Subjective symptoms were provided by conscious, cognitively and mentally normal, and linguistically competent to respond to the interview: patients, health care clinicians, and their families. All keywords were provided to the statisticians by two trained and board-certified physiatrists. Neurologic manifestations were categorized into central nervous system (CNS) manifestations (dizziness, headache, impaired consciousness, acute cerebrovascular disease, ataxia, and seizure) and peripheral nervous system (PNS) manifestations (taste impairment, smell impairment, vision impairment, and nerve pain).

Statistical analysis

The Chi-square test or Fisher's exact test was used when appropriate to examine the differences between CNS and non-CNS and between PNS and non-PNS in categorical variables. The differences in continuous variables were analyzed using the Mann-Whitney U test to compare the group of patients with CNS or PNS to those without CNS or PNS. Analyses were done using the software SAS 7.1 (SAS Institute Inc., Cary, NC). All tests were two-sided. A value of p < 0.05 was considered statistically significant. 

## Results

Demographic and clinical characteristics

A total of 5,455 hospitalized patients with confirmed COVID-19 infection were included in the analysis. Their demographic and clinical conditions are shown in Table 1. Their mean (SD) age was 58 years. Out of the total, 3,419 were males (62.7%), and 2036 were females (37.3%); 2,325 were Hispanics (49.7%), and 2,353 (50.3%) were non-Hispanics. Since Hispanics heavily populate the two boroughs of New York City, we chose to do a comparative analysis. However, we found no statistical significance.

**Table 1:** Demographics and comorbidities of patients admitted with a diagnosis of COVID-19 COVID+: COVID-19 positive patients; CNS: patients with Central Nervous system involvement; Non-CNS: Patients without Central Nervous System  involvement

	COVID+ Patients (N = 5455)		Non-CNS (N = 5170)		CNS (N = 285)		P-value
	N	%	N	%	N	%	
Demographics							
Age, median/ SD	58.0/ 17.3		58.0 /17.4		60.0 /16.0		
Age,							0.0416
18 - <50	1774	32.5	1697	32.8	77	27.0	
>=50	3681	67.5	3473	67.2	208	73.0	
Gender							0.4228
Female	2036	37.3	1936	37.5	100	35.1	
Male	3419	62.7	3234	62.6	185	64.9	
Ethnicity							
Hispanic	2325	49.7	2206	49.8	119	47.6	0.4947
Non-Hispanic	2353	50.3	2222	50.2	131	52.4	
Unknow/Decline/Other	1027, 18.8% (excluded from the analysis)						
Health History							
Hypertension	2528	46.3	2356	45.6	172	60.4	< .0001
Chronic Kidney Disease	355	6.5	319	6.2	36	12.6	< .0001
Congestive Heart Failure	300	5.5	273	5.3	27	9.5	<0.1
Coronary Artery Disease	373	6.8	351	6.8	22	7.7	0.5447
Malignancy	13	0.2	13	0.3	0	0.0	1.0000
Cerebrovascular Disease	66	1.2	58	1.1	8	2.8	0.0113
Diabetes	1761	32.3	1650	31.9	111	39.0	0.0134
Anorexia	6	0.1	6	0.1	0	0.0	1.0000
Any	3062	56.1	2868	55.5	194	68.1	< .0001

Typical symptoms at the time of presentation

The most common symptoms at the onset of illness were fever 1,014 (18.2%), cough 943 (16.9%), abdominal pain 130 (2.3%), diarrhea 39(0.7%), and anorexia 6, (0.1%). Fever and upper respiratory symptoms predominated (Table 2).

**Table 2:** Typical Symptoms at the time of presentation COVID+: COVID-19 positive patients; CNS: patients with Central Nervous system involvement; Non-CNS: Patients without Central Nervous System  involvement

	COVID+ Encounters (N = 5571)		Non-CNS (N = 5276)		CNS (N = 295)		P Value
	N	%	N	%	N	%	
Typical Symptoms							
Diarrhea	39	0.7	39	0.7	0	0.0	0.2682
Fever	1014	18.2	995	18.9	19	6.4	<0.0001
Cough	943	16.9	930	17.6	13	4.4	<0.0001
Throat Pain	0	0	0	0	0	0	
Abdominal Pain	130	2.3	125	2.4	5	1.7	0.4553

Of the 5,455 patients, 285 patients (5.2%) had nervous system manifestations with a prevalence in people older than 60 (p-value 0.0416.) In patients with CNS manifestations, the predominant presenting symptom was headache seen in 88 (1.5%). The seizure was the next in 75 (1.3%). Encephalitis was the presenting symptom in 59 (1.0%). Altered mental status combined with impaired consciousness was seen in a total of 15 (0.2%). Peripheral nervous system findings such as anosmia, ageusia, muscle weakness, and numbness were seen in 0.4%. 

Laboratory findings in patients with and without CNS manifestations

Table 3 shows the laboratory findings in CNS and non-CNS subgroups. Patients with CNS symptoms had increased creatinine levels of 1.1 mg/dl (p-value 0.01), D-dimer levels of 684.5 ng/ml (p-value 0.0062), and lactate dehydrogenase (LDH) levels of 2.3 mmol/l (p-value of 0.0394) compared to the 5,276 patients without CNS manifestations. Multiorgan failure was common in these patients.

**Table 3:** Significant lab characteristics at the time of admission CNS: Central nervous system; BUN: Blood urea nitrogen, CRP: C-reactive protein: Abs count: Absolute count; WBC: White blood cells; D-Dimer: Fibrin degradation product

	Median (Range)			
	COVID+ Encounters (N = 5571)	Non-CNS (N = 5276)	CNS (N = 295)	p-Value
Lab Characteristics				
BUN (mg/dl)	16 (3-329)	16 (3-329)	17 (4-264)	0.0644
Creatinine (mg/dl)	1.0 (0.2-32.2)	1.0 (0.2-32.2)	1.1 (0.3-26.1)	0.0141
CRP (mg/l)	84.4 (0.1-668.3)	85.1 (0.1-668.3)	73 (1.9-465.4)	0.3426
D-Dimer (ng/ml)	538.5 (150-76151)	531 (150-76151)	684.5 (164-52303)	< .01
Lactate Dehydrogenase (mmol/l)	2.1 (0.4-26)	2.1 (0.4-26)	2.3 (0.7-12)	< .05
Lymphocytes Abs Count (nl)	ALL NULL			
Platelets (nl)	215 (7-885)	216 (7-885)	209 (39-771)	0.0769
Neutrophil Abs (nl)	6.6 (0.2-32.9)	6.6 (0.2-32.9)	7.6 (0.5-22.7)	0.2897
WBC (nl)	7.7 (0.2-226.7)	7.7 (0.2-226.7)	7.6 (2.2-34.2)	0.9477

Comorbidities

Of these 5,455 patients, 2,528 (46.3%) had hypertension (HTN), 1,761 (32.3%) had diabetes (DM), 355 (6.5%) had chronic kidney disease (CKD), 373 (6.8%) had coronary artery disease (CAD), 300 (5.5%) had congestive heart failure (CHF), 13 (0.2%) had malignancy, and 66 (1.2%) had cerebrovascular disease (CVA). CNS manifestations were found in 285 (5.2%) and out of the total patients with CNS manifestations, 172 (60.4%, p-value 0.001) had hypertension, 36 (12.6%, p-value 0.001) had CKD. CHF was seen in 27 (9.5%, p-value 0.0025 ). Other significant findings were CVA in 8 (2.8%, P-value 0.0113). Diabetes was found in 111 (39%, p-value 0.0134). Significant p-values in patients with CNS manifestations are shown in Table 4.

**Table 4:** Neurological manifestations associated with COVID-19 CNS: Central Nervous system; PNS: Peripheral Nervous System; NR: Not reported. Comparison of findings of various authors. The neurological symptoms are listed. Pinna et al. [4]; Xiong et al. [5]

	Studies		
Clinical Feature of Diagnosis	Present study	Pinna et al.	Xiong et al.
Total patients with COVID-19	5455	650	917
Patients with CNS manifestations	285 (5.2%)	50 (7.7%)	39 (4.2%)
Headache	75 (1.3%)	12 (24%)	2 (5.1%)
Dizziness	47 (0.8%)	NR	NR
Impaired Consciousness	15 (0.2%)	30 (60%)	25 (64.1%)
Stroke	66 (1.2%)	20 (40%)	10 (25.6%)
Seizures	75 (1.3%)	13 (26%)	0 (0.0%)
Patients with PNS manifestations	20 (0.4%)	NR	NR
Encephalitis	59 (1.1%)	NR	0 (0.0%)

Patients older than 50 years of age and had at least one comorbidity seemed to be more prone to develop CNS findings. The most common comorbidity was HTN, followed by DM, CKD, CHF, and CVA. Table 5 shows a comparison of neurological manifestations associated with COVID-19 found by various authors. 

**Table 5:** Variables significantly correlated with CNS manifestations CNS: Central Nervous system

	P Value
Age, y	0.0416
Hypertension	< .0001>
Chronic Kidney Disease	< .0001>
Congestive Heart Failure	0.0025
Cerebrovascular Disease	0.0113
Diabetes	0.0134
Any Variable	< .0001>
Fever	<0.0001
Cough	<0.0001
Admission Creatinine	0.0141
Admission D-Dimer	0.0062
Admission Lactate Dehydrogenase	0.0394

Our literature review found two publications whose results correlated with ours. In table 5, we summarized those findings and included our findings. COVID-19 with neurological manifestations was found in 7.7% in Pinna's series [4], 4.2 % in the Xiong [5] series, and 5.2%. in ours. We agree with these results. The risk of critical neurologic events was highly associated with age above 50 years and previous comorbidities and neurologic conditions.

## Discussion

The COVID-19 outbreak in Wuhan, China quickly became a pandemic and posed several epidemiological, social, and medical issues. The healthcare systems and governments worldwide were forced to take extreme measures to contain the infection and develop treatments. The first 85 cases were reported in the epicenter of the outbreak, the city of Wuhan in Hubei province, China [2]. The median age of the patients was 59 years with higher morbidity and mortality among the elderly and those with coexisting conditions (similar to the situation with influenza); 56% of the patients were male.

A SARS-CoV-2 virus is spread predominantly via respiratory droplets from symptomatic and asymptomatic infected individuals. The most common symptoms of COVID-I9 are fever and dry cough. Rhinorrhea and gastrointestinal symptoms are much less frequent. Most patients (81%) have mild symptoms with no pneumonia or mild pneumonia, 14% have severe respiratory distress, and 5% have respiratory failure, Septic shock, and multiorgan failure.

The neurologic symptoms can be categorized into CNS (Central Nervous System) manifestations and PNS (Peripheral Nervous System) manifestations. CNS symptoms vary from headache, dizziness, headache, ageusia, anosmia, and seizures to frank strokes. They seem to occur more in hospitalized patients who are elderly and are severely sick. PNS manifestations included musculoskeletal symptoms such as myalgia, cramps, tingling, and numbness in the extremities. An interesting finding of Guillian Barré' syndrome (GBS) and encephalitis was reported by Helbok et al. [6] even in those who lacked the typical pulmonary symptoms and cough.

Biological mechanisms

Huang et al. described the SARS-CoV-2 as a novel single-stranded enveloped RNA virus among the seven known human coronaviruses [7]. It was recognized in December 2019. According to Wang et al., this virus is structurally related to severe acute respiratory syndrome (SARS) like many other coronaviruses [8]. SARS-CoV-2 is believed to have originated in bats, as it shares 79.5% genome sequence identity with SARS-Cov and 89-960/o nucleotide identity with bat coronaviruses [9].

Angiotensin-converting enzyme 2 (ACE 2) has been identified as the functional receptor for COVID-19, which is present in the human nervous system and skeletal muscle. COVID-19 may enter the human body through the retrograde neuronal or the hematogenous route. Autopsy studies have also concluded that the virus may be present in the cerebral spinal fluid and brain tissue. Patients with severe Infection showed higher D-dimer levels and were more likely to develop cerebrovascular diseases. 

Both SARS and COVID-19 are micro-invasive and neurotropic. Both SARS-CoV-2 and SARS-COV enter human cells via the ACE2 receptor 26, an essential component of the renin-angiotensin system in the brain. Therefore, like SARS-COV, SARS-CoV-2 might also invade the CNS. The mode of replication of SARS-CoV-2 is like those of micro-invasive animal coronaviruses.

Two theories have been proposed by Wu et al. [10] and Desforges et al. [11] for neural invasion by the human coronaviruses: hematogenous route and neuronal dissemination. Coronaviruses pass through the epithelial barrier, gain access to the bloodstream, and enter the CNS by either infecting endothelial cells of the blood-brain barrier or epithelial cells of the blood-CSF barrier in the choroid plexus. Infected leukocytes act as vectors for the transportation of the virus into the brain. Pellegrini et al. proposed a neuroinflammatory state as a cause of the significant breakdown of the brain-CSF barrier [12]. The authors propose that the leakiness of the barrier could promote the entry of inflammatory cytokines into the CNS.

Another possible mechanism is neuronal dissemination as described by Berth et al. [13]. Non-human viruses invade peripheral nerve terminals, spread retrogradely across nerve synapses, and thus access the CNS. Brain injury was shown to occur via the olfactory pathway. Kanberg et al. [14] propose several mechanisms for neuronal invasion by COVID-1. Among these direct effects of viral infection, primarily in the olfactory mucosa; indirect effects of the systemic inflammatory response leading to activation of CNS-resident immune cells; microvascular injury and thrombosis related to the hypercoagulable state, as well as endotheliitis resulting from viral interaction with perivascular cells; and other unspecific hypoxic/toxic consequences of severe disease leading to CNS immune activation. Further spread to the cardiorespiratory centers of the medulla oblongata is believed to be the cause of the subsequent death as described by McCray et al. [15]. Lukiw et al. [16] stated that two CNS blood plasma markers have been identified: (i) NfL (neurofilament light chain protein), a marker of intra-axonal neuronal injury) and (ii) GFA p (glial fibrillary acidic protein), a marker of astrocytic activation/injury. These markers are elevated in patients with severe disease.

In summary, CNS damage is a prominent feature in moderate to severe COVID-19 disease; however, it is a consequence of direct neurotropism and the neuro damaging mechanism of SARS-CoV-2.

COVID-19 Infection is associated with vascular thrombotic complications, frequently seen in severely ill patients and indicates a poor prognosis according to Tang et al. [17]. Despite prophylactic anticoagulation, 8 to 31% of patients developed deep vein thrombosis (DVT), pulmonary embolism (PE), venous thromboembolism (VTE), and acute ischemic strokes (AIS). Lodigiani et al. reported that VTE incidence was more than five times that compared to control groups with non-COVID-19 acute respiratory distress syndrome (ARDS) [18]. The patients with cerebrovascular diseases have a high inflammatory response and abnormal coagulation with elevated C-reactive protein and D-dimer levels. The patients were about 70 years of age. However acute ischemic strokes have been described by Oxley et al. [19]. A case series from New York described five SARS-CoV-2 positive patients under 58 years with large vessel stroke by González-Pinto et al. [20].

CNS manifestations

Symptom manifestations vary from altered mental status to cardiovascular accidents. Studies indicate that as COVID-19 progresses, neurological manifestation in inpatient populations may also increase. Various case reports and case series have found a relationship between COVID-19 patients and neurologic manifestations. The initial case series came from Wuhan, China where Mao et al. [3] observed a link between patients and neurological symptoms in 78 out of 214 COVID-19 patients (36.4%). Romero-Sanchez et al. detected neurological symptoms in 484 of 841 (57.5%) COVID patients in Spain [21]. Karadas et al. detected neurological symptoms in 83 of 239 COVID patients (34.7%) in Chicago [22]. Pinna et al. reported a 7.7 % incidence [4]. This wide variability may be due to the patient demographics and reporting styles.

The ability to accurately evaluate the connection between CNS manifestation and COVID-19 was limited because the focus was to find a cure at the pandemic's start. Understanding the link between CNS manifestations was not a primary focus, and therefore certain factors limited early studies. Limitation factors included examinations, documentation, high mortality rates, and many studies were based on electronic medical records resulting in incorrect collection and analysis of neurologic findings. In addition, many recorded symptoms were based on subjective descriptions provided by patients, which often serves as the basis for the misrepresentation. 

Neurological symptoms are a feature of COVID-19 Infection. They vary from anosmia and ageusia to more severe complications such as stroke and encephalitis. The potential mechanism discussed needs further research and a much more thorough study. It is not yet clear if cognitive deficits will present as long-term consequences of CNS involvement.

Stroke

Acute brain ischemia is a frequent complication of the COVID-19 pandemic. Viral infections are known to activate a coagulation cascade; COVID-19 patients have a very high incidence of induced coagulopathy. It has been described in 21.6% of patients with severe COVID-19 and is highly associated with a poor prognosis. Coagulopathy is characterized by elevated prothrombin time, D-dimer levels, and thrombocytopenia but without hypofibrinogenemia. Al-Ani et al. [23] and Iba et al. [24] suggest that coagulopathy is related to an infection-induced systemic inflammatory response involving endothelial dysfunction and micro thrombosis with organ failure and usually no bleeding. Also, Wichmann et al. [25] in their autopsy studies found that disseminated intravascular coagulation (DIC) is frequently detected in deceased patients diagnosed with COVID-19. D-dimer values reflect disease severity and the consumptive coagulation system. It is characterized by abnormally increased procoagulant pathways, resulting in intravascular fibrin deposition and decreased hemostatic components including platelets, fibrinogen, and other clotting factors. Although chronic DIC can be asymptomatic, acute DIC results in bleeding and intravascular thrombus formation, leading to tissue hypoxia, multiorgan dysfunction, and death.

Yaghi et al., in a New York health care system report, said that the risk factors and underlying mechanisms of stroke are utterly different in patients with COVID-19 and general strokes [26]. Some of these patients were young and did not have a history of HTN.

In addition, Pons et al. describe the vascular endothelium as the cornerstone of organ dysfunction in severe SARS-CoV-2 infection [27]. SARS-CoV-2 can infect and injure endothelial cells, leading to systemic arterial and venous microvascular and macrovascular complications. Capillary thrombosis can lead to multiorgan failure. High creatinine and lactate dehydrogenase (LDH) indicate kidney and liver damage as part of the multiorgan failure.

PNS manifestations

Although not seen in our series, Guillain Barre syndrome (GBS) was found in five patients among 1,000-1,200 patients affected with COVID-19 as reported by Toscano et al. in Northern Italy [28]. Another study by Willison et al. supports the finding that the Incidence of GBS in patients with COVID-19 seems to be much higher than the expected Incidence in the general population, which is 0·8-1·9 cases per 100,000 persons per year [29]. It is unclear if one can conclude that the SARS-CoV-2 might induce immune-mediated damage to the nerves after a latent period following the infection as reported by Chen et al. [30] in a descriptive study published in the Lancet.

Limitations of the current study

There are several limitations to this study. First, this is a retrospective analysis and lacks standardization. What would be defined as CNS-related neurological symptoms under normal circumstances, i.e., unilateral weakness, numbness, aphasia, cranial nerve involvement, and cognitive symptoms, were challenging to assess. Secondly, many of these patients rapidly deteriorated, and life-saving measures took precedence. Many remained unconscious for a long time due to their metabolic instability or induction of sedation for ventilation requirements. Thus, communication was difficult. Third, since the data from most of these studies were extracted from electronic medical records retrospectively, documentation variations could have played a role. Fourth, due to significant contamination risk, studies such as scanning, MRI, and lumbar puncture had to be limited. Fifth, non-specific symptoms, such as headache, dizziness, fatigue, and myalgia, could be related to systemic conditions.

## Conclusions

In summary, we reviewed 5,455 patients from four inner-city New York Hospitals, admitted with COVID-19. Of these, 285 patients were found to have CNS manifestations. We analyzed this data to prognosticate CNS involvement factors. We found that patients above 50 years of age are at risk for developing neurological manifestations. We observe that even though the involvement is not high, patients with multiple comorbidities are at higher risk for developing neurologic manifestations. Several possible mechanisms play a role in the clinical observations. SARS-CoV-2 may enter the CNS through the hematogenous or retrograde neuronal route. Deficits with smell could be related to retrograde neuronal transport.

Elevated levels of D-dimer in patients with CNS symptoms indicate the effects of DIC deficits leading to cerebral hemorrhage. Patients with DIC may develop cerebrovascular diseases. Although not very high, some patients showed evidence of AIDP (acute inflammatory demyelinating polyneuropathy), which may be related to post-infectious, immune-mediated complications,

As the outbreak continues to spread, and more and more novel variants of the virus are emerging, an increase in the incidence of neurologic manifestations is a possibility. Future studies need to be carefully designed, taking into account several methodological issues. Clinicians should be aware that there is evidence that encephalitis, encephalopathy, and cerebrovascular incidents are possible in patients with COVID-19. This requires CSF examination, neurophysiological studies, spinal imaging; brain digital subtraction angiography, or cerebral vessel wall imaging in patients with presumed cerebral vasculitis. A high clinical suspicion should be exercised in patients who present with or develop CNS symptoms so that the necessary diagnostic workup can be initialized, to avoid any delays. and contribute to poor prognosis.

At this time, the future possibilities for and long-term consequences of this virus are unclear. The potential for longitudinal neurological consequences such as demyelinating disorders, cognitive deficits, muscle weakness, strokes, and myalgias have to be studied. To date, there are no studies available explicitly addressing the long-term cognitive effects of this virus. Future studies should consider neuroimaging, electrophysiologic studies, lab, and neuropsychological evaluations to examine CNS damage and neurological alterations.

## References

[1] Thompson CN, Baumgartner J, Pichardo C (2020 ). COVID-19 outbreak — New York City, February 29-June 1, 2020. MMWR Morb Mortal Wkly Rep 2020.

[2] Du Y, Tu L, Zhu P (2020). Clinical features of 85 fatal cases of COVID-19 from Wuhan. A retrospective observational study. Am J Respir Crit Care Med.

[3] Mao L, Jin H, Wang M (2020). Neurologic manifestations of hospitalized patients with coronavirus disease 2019 in Wuhan, China. JAMA Neurol.

[4] Pinna P, Grewal P, Hall JP (2020). Neurological manifestations and COVID-19: experiences from a tertiary care center at the frontline. J Neurol Sci.

[5] Xiong W, Mu J, Guo J (2020). New onset neurologic events in people with COVID-19 in 3 regions in China. Neurology.

[6] Helbok R, Beer R, Löscher W (2020). Guillain-Barré syndrome in a patient with antibodies against SARS-COV-2. Eur J Neurol.

[7] Huang C, Wang Y, Li X (2020). Clinical features of patients infected with 2019 novel coronavirus in Wuhan, China. Lancet.

[8] Wang D, Hu B, Hu C (2020). Clinical characteristics of 138 hospitalized patients with 2019 novel coronavirus-infected pneumonia in Wuhan, China. JAMA.

[9] Guan WJ, Ni ZY, Hu Y (2020). Clinical characteristics of coronavirus disease 2019 in China. N Engl J Med.

[10] Wu C, Chen X, Cai Y (2020). Risk factors associated with acute respiratory distress syndrome and death in patients with coronavirus disease 2019 pneumonia in Wuhan, China. JAMA Intern Med.

[11] Desforges M, Le Coupanec A, Dubeau P, Bourgouin A, Lajoie L, Dubé M, Talbot PJ (2019). Human coronaviruses and other respiratory viruses: underestimated opportunistic pathogens of the central nervous system?. Viruses.

[12] Pellegrini L, Albecka A, Mallery DL (2020). SARS-CoV-2 infects the brain choroid plexus and disrupts the blood-CSF barrier in human brain organoids. Cell Stem Cell.

[13] Berth SH, Leopold PL, Morfini GN (2009). Virus-induced neuronal dysfunction and degeneration. Front Biosci (Landmark Ed).

[14] McCray PB Jr, Pewe L, Wohlford-Lenane C (2007). Lethal infection of K18-hACE2 mice infected with severe acute respiratory syndrome coronavirus. J Virol.

[15] Kanberg N, Ashton NJ, Andersson LM (2020). Neurochemical evidence of astrocytic and neuronal injury commonly found in COVID-19. Neurology.

[16] Lukiw WJ, Pogue A, Hill JM (2022). SARS-CoV-2 infectivity and neurological targets in the brain. Cell Mol Neurobiol.

[17] Tang N, Bai H, Chen X, Gong J, Li D, Sun Z (2020). Anticoagulant treatment is associated with decreased mortality in severe coronavirus disease 2019 patients with coagulopathy. J Thromb Haemost.

[18] Lodigiani C, Iapichino G, Carenzo L (2020). Venous and arterial thromboembolic complications in COVID-19 patients admitted to an academic hospital in Milan, Italy. Thromb Res.

[19] Oxley TJ, Mocco J, Majidi S (2020). Large-vessel stroke as a presenting feature of Covid-19 in the young. N Engl J Med.

[20] González-Pinto T, Luna-Rodríguez A, Moreno-Estébanez A, Agirre-Beitia G, Rodríguez-Antigüedad A, Ruiz-Lopez M (2020). Emergency room neurology in times of COVID-19: malignant ischaemic stroke and SARS-CoV-2 infection. Eur J Neurol.

[21] Romero-Sánchez CM, Díaz-Maroto I, Fernández-Díaz E (2020). Neurologic manifestations in hospitalized patients with COVID-19: The ALBACOVID registry. Neurology.

[22] Karadaş Ö, Öztürk B, Sonkaya AR (2020). A prospective clinical study of detailed neurological manifestations in patients with COVID-19. Neurol Sci.

[23] Al-Ani F, Chehade S, Lazo-Langner A (2020). Thrombosis risk associated with COVID-19 infection. A scoping review. Thromb Res.

[24] Iba T, Levy JH, Warkentin TE, Thachil J, van der Poll T, Levi M (2019). Diagnosis and management of sepsis-induced coagulopathy and disseminated intravascular coagulation. J Thromb Haemost.

[25] Wichmann D, Sperhake JP, Lütgehetmann M (2020). Autopsy findings and venous thromboembolism in patients with COVID-19: a prospective cohort study. Ann Intern Med.

[26] Yaghi S, Ishida K, Torres J (2020). SARS-CoV-2 and stroke in a New York healthcare system. Stroke.

[27] Pons S, Fodil S, Azoulay E, Zafrani L (2020). The vascular endothelium: the cornerstone of organ dysfunction in severe SARS-CoV-2 infection. Crit Care.

[28] Toscano G, Palmerini F, Ravaglia S (2020). Guillain-Barré Syndrome associated with SARS-CoV-2. N Engl J Med.

[29] Willison HJ, Jacobs BC, van Doorn PA (2016). Guillain-Barré syndrome. Lancet.

[30] Chen N, Zhou M, Dong X (2020). Epidemiological and clinical characteristics of 99 cases of 2019 novel coronavirus pneumonia in Wuhan, China: a descriptive study. Lancet.

